# Supplementary material for: Action Observation for Children and Adolescents with Cerebral Palsy: Hope or Hype? A Systematic Review with Meta-Analysis
Source: Children (Basel). 2025 Jun 20;12(7):810. doi: 10.3390/children12070810 (PMC12293779; doi:10.3390/children12070810)

Supplementary Material.

Supplementary Material – Table S1 – Search engines, databases, search equations and results.

Search Engine	Databases	Searches (nº)	Original search		Actualization search (New registries – Original)	
			**Equation**	**Registries *(date)***	**Equation**	**Registries *(date)***
PubMed	MEDLINE	Nº1	(“Cerebral Palsy”[Mesh] OR “cerebral palsy”[Title/Abstract]) AND (“action observation”[Title/Abstract] OR “skill observation”[Title/Abstract] OR “movement observation”[Title/Abstract] OR “task observation”[Title/Abstract] OR “motor observation”[Title/Abstract]) AND “random*”[Title/Abstract]	23 *(12th Apr 2022)*	("Cerebral Palsy"[Mesh] OR "cerebral palsy"[Title/Abstract]) AND ("action observation"[Title/Abstract] OR "skill observation"[Title/Abstract] OR "movement observation"[Title/Abstract] OR "task observation"[Title/Abstract] OR "motor observation"[Title/Abstract]) AND "random*"[Title/Abstract] AND 2022/04/13:2024/07/22[dp]	8 *(22th July 2024)*
Web of Science	Web of Science Core Collection; Current Contents Connect; Derwent Innovations Index; KCI-Korean Journal Database; MEDLINE; and SciELO Citation Index.	Nº1	(TS=(“cerebral palsy”) AND TS=(“trial”) AND (TS=(“action observation”) OR TS=(“skill observation”) OR TS=(“movement observation”) OR TS=(“task observation”) OR TS=(“motor observation”)))	23 *(12th Apr 2022)*	(TS=(“cerebral palsy”) AND TS=(“trial”) AND (TS=(“action observation”) OR TS=(“skill observation”) OR TS=(“movement observation”) OR TS=(“task observation”) OR TS=(“motor observation”)))<br>*Publication date range: 2022-04-13 to 2024-07-22*	5 *(22th July 2024)*
EBSCO	Academic Search Premier; Education Source; ERIC; Library, Information Science & Technology Abstracts; MEDLINE Complete; OpenDissertations; PSICODOC; Sociology Source Ultimate; Teacher Reference Center; and The Serials Directory	Nº1	TI “Cerebral palsy” AND AB “trial” AND (AB “action observation” OR AB “skill observation” OR AB “movement observation” OR AB “task observation” OR AB “motor observation”)	12 *(12th Apr 2022)*	TI “Cerebral palsy” AND AB “trial” AND (AB “action observation” OR AB “skill observation” OR AB “movement observation” OR AB “task observation” OR AB “motor observation”)<br>*Publication date range: 2022-04 to 2024*	3 *(22th July 2024)*
EMBASE	EMBASE	Nº1	“cerebral palsy”:ti AND “action observation”:ab AND “trial”:ab.	12 *(7th Apr 2022)*	**Not available access**	-
Cochrane Central	Cochrane Central	Nº1	“Cerebral palsy”:ti,ab,kw AND (“action observation” OR “skill observation” OR “movement observation” OR “task observation” OR “motor observation”):ti,ab,kw.	37 *(12th Apr 2022)*	“Cerebral palsy”:ti,ab,kw AND (“action observation” OR “skill observation” OR “movement observation” OR “task observation” OR “motor observation”):ti,ab,kw.<br>*Publication date range: 2022-04-13 to 2024-07-22*	22 *(22th July 2024)*
Google scholar	Google scholar	Nº1	(“action observation” OR “skill observation” OR “movement observation” OR “task observation” OR “motor observation”) + “randomized” + intitle:“cerebral palsy” + intitle:“trial”<br>*Not including citations nor patents*	22 *(12th Apr 2022)*	(“action observation” OR “skill observation” OR “movement observation” OR “task observation” OR “motor observation”) + “randomized” + intitle:“cerebral palsy” + intitle:“trial”<br>*Not including citations nor patents*<br>*Publication date range: 2022 to 2024*	13 *(22th July 2024)*
		Nº2	“observación de acciones” AND “ensayo” AND intitle: “parálisis cerebral”	1 *(7th Apr 2022)*	(“observación de acciones” OR "observación de acciones") + “ensayo” + (intitle:“paralisis cerebral” OR intitle:"parálisis cerebral")<br>*Not including citations nor patents*<br>*Publication date range: 2022 to 2024*	3 *(22th July 2024)*

PEDro	PEDro	Nº1	Abstract & Title: cerebral palsy action observation	7 clinical trials *(7th Apr 2022)*	Abstract & Title: cerebral palsy action observation<br>*New records added since: 07/04/2022*	0 *(22th July 2024)*

Supplementary Material – Figure S1 – Funnel plot of unilateral upper limb function meta-analysis.

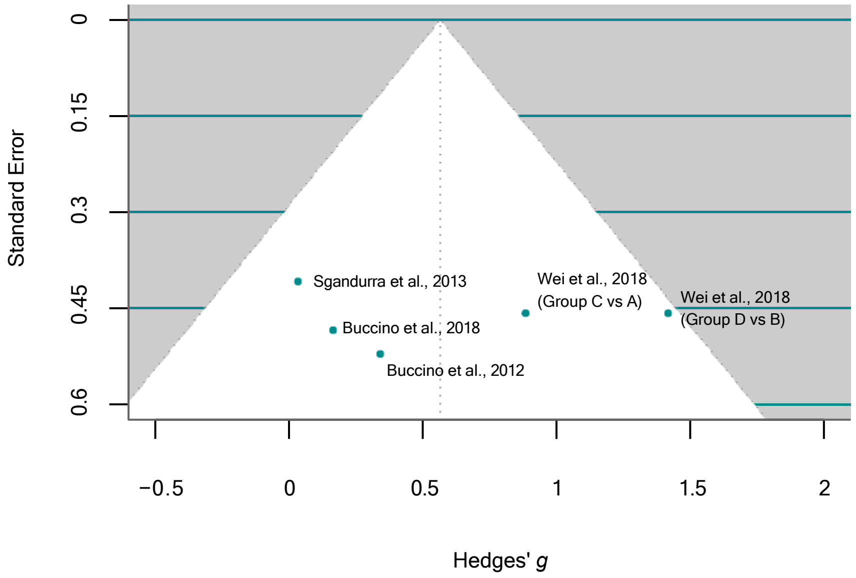

Supplementary Material – Figure S2 – Funnel plot of assisting hand ability during bimanual activities meta-analysis.

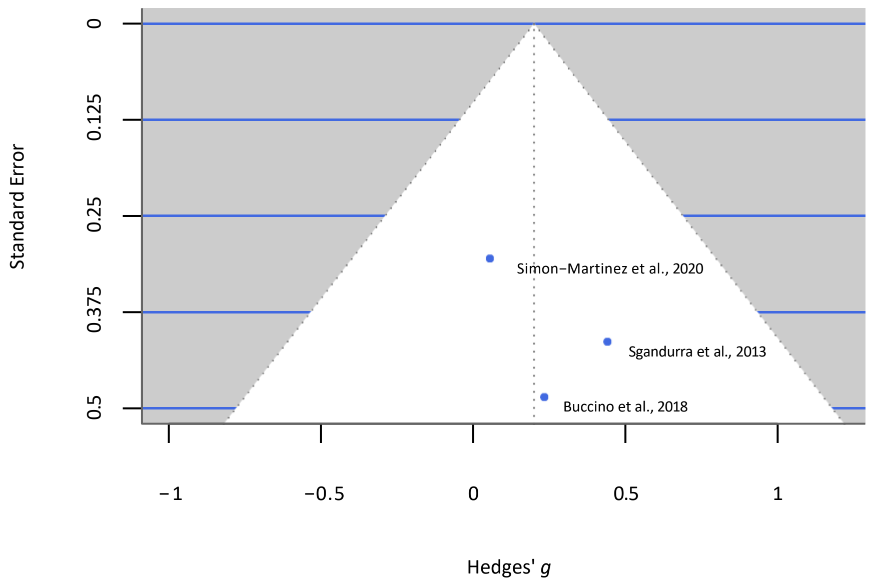

Supplementary Material – Figure S3 – Funnel plot of manual function during daily activities meta analysis

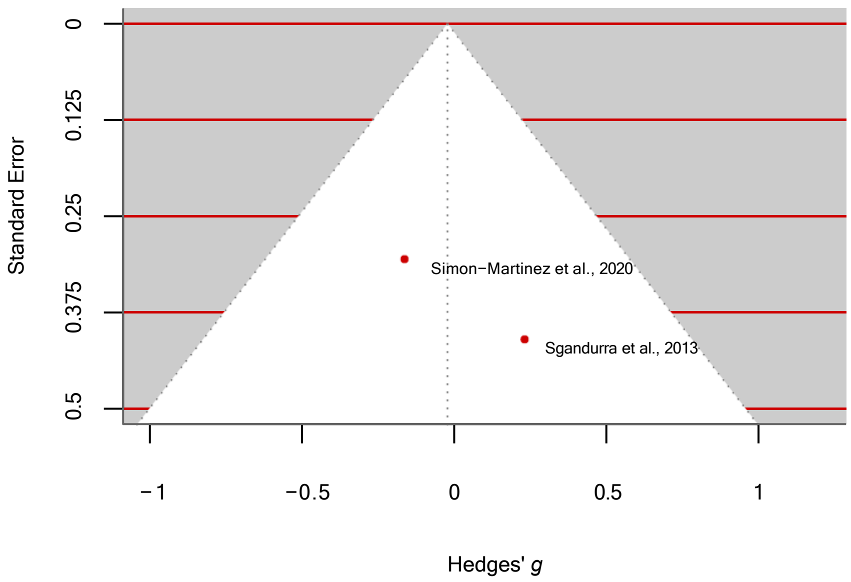

Supplementary Material – Figure S4 – Funnel plot of hand grip strength meta analysis

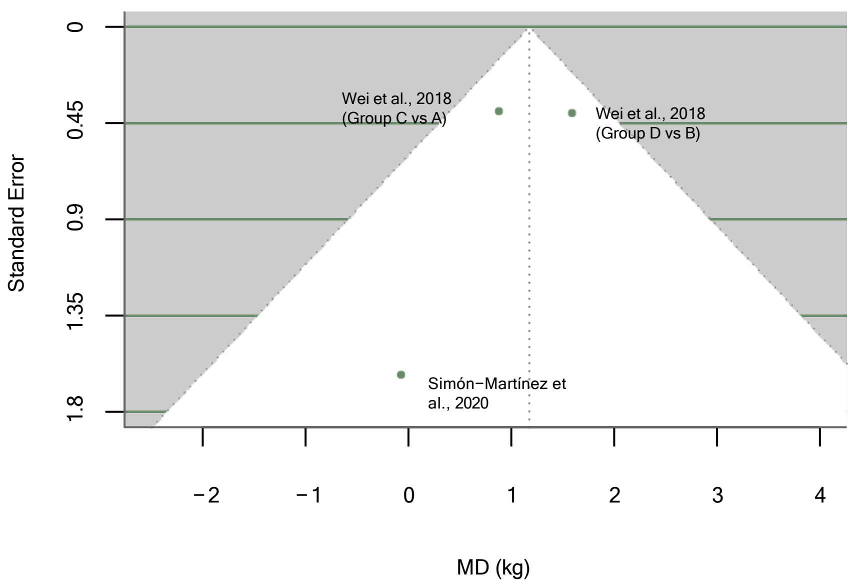

Supplementary Material – Figure S5 – Funnel plot gross motor function standing dimension meta analysis

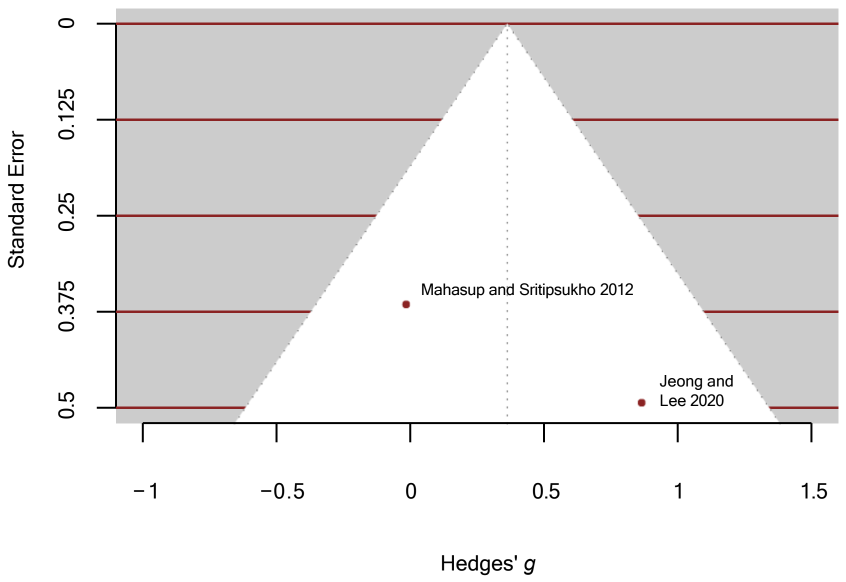

Supplementary Material – Figure S6 – Funnel plot gross motor function walking, standing and jumping dimensions, dimension meta analysis.

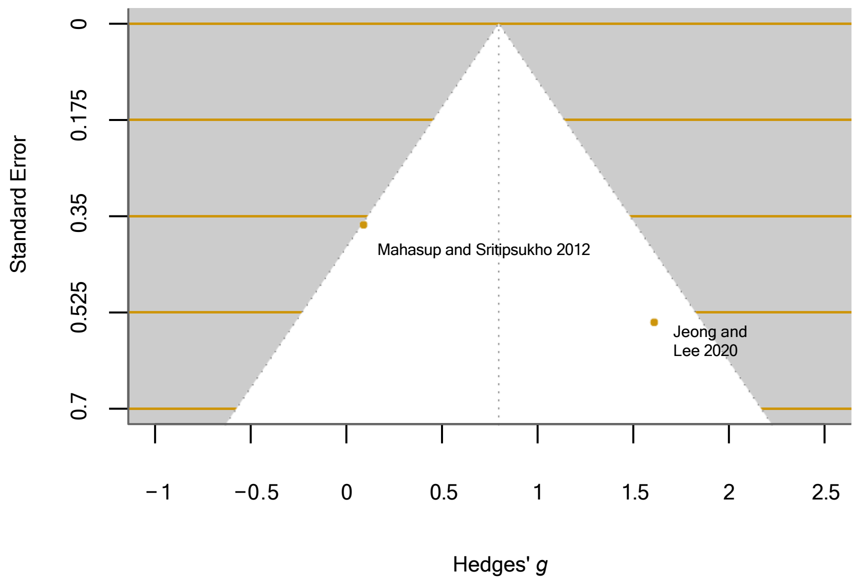

Supplement: Supplementary file 1 [file children-12-00810-s001.zip › children-3682906-supplementary.pdf]
